# Care of the Feet

**Published:** 1877-06

**Authors:** 


### CARE OF THE FEET.

One evening in Boston, just as *Washington Alston*, the Painter, was approaching the door of a dwelling, where a splendid party had assembled, he suddenly stopped short and said to his friend,

"I cannot go in."

Nonsense! why not?

"I have a hole in one of my stockings."

Pshaw, man, nobody knows it.

"But I do," said the celebrated Artist.

